# Postsurgical Pyoderma Gangrenosum Following Carpal Tunnel Release: A Rare Disease Following a Common Surgery

**Published:** 2017-03-21

**Authors:** Michael R. Ruebhausen, Shaun D. Mendenhall, Michael W. Neumeister, Nada N. Berry

**Affiliations:** Institute for Plastic Surgery, Southern Illinois University School of Medicine, Springfield, IL

**Keywords:** pyoderma gangrenosum, carpal tunnel, necrotizing infection, postsurgical, nonhealing wound

## Abstract

**Objective:** Postsurgical pyoderma gangrenosum is a rare but potentially devastating condition for surgical patients. While pyoderma gangrenosum has 2 subtypes, typical and atypical, each can be further classified by its heralding features. These include ulcerative, pustular, bullous, and vegetative. The presentation can be a result of trauma or, as mentioned before, postsurgical. The plastic and reconstructive surgeon most likely will encounter postsurgical pyoderma gangrenosum in practice, as it has been reported in patient populations frequently seen in plastic surgery clinics. **Methods:** We present a unique case of idiopathic postsurgical pyoderma gangrenosum in a patient who presented after carpal tunnel release, the most common surgery of the hand and wrist performed in the United States annually. This is believed to be the first ever case reported in the literature of pyoderma gangrenosum following carpal tunnel release. **Results:** The patient's disease course was complicated by surgical debridement prior to diagnosis. Unfortunately, this condition necessitated mid-forearm amputation. The wound eventually healed with primary closure and appropriate medical therapy. **Conclusion:** Previous experience with this disease, a high index of suspicion, and general education regarding the disease process and its management could potentially have prevented this outcome. We hope to underscore that it is important to consider a patient's entire history and to have a high index of suspicion in unusual postsurgical wounds in order to adequately diagnose, treat, and manage patients who develop postsurgical pyoderma gangrenosum.

The literature abounds with information regarding treatment of pyoderma gangrenosum (PG); however, much of this is published in the medical and dermatologic literature. Much of the published literature in the surgical community comes from small case reports and limited literature reviews, which, due to the rarity of the disease, do not encompass the numerous forms of PG. Pyoderma gangrenosum is a rare disease that presents in 2 subtypes: typical and atypical. The typical form is often deeper and ulcerative. It is most commonly associated with inflammatory bowel disease,^1^ especially ulcerative colitis, but can also be seen with other autoimmune disease such as rheumatoid arthritis.^2^ The atypical disease is less commonly associated with other disease processes, although a recent review of the literature demonstrated that in more than 50% of cases, there is an identifiable predisposing factor in the patient's medical history that is likely linked to the development of PG. In the setting of these predisposing factors, there are several inciting factors.^3^^,^^4^

Surgery, for instance, is known to cause PG ulceration. The same literature review found that 25% of the time when PG was noted postoperatively, termed postsurgical pyoderma gangrenosum (PSPG), it was after a breast surgery.^4^ A high percentage of these individuals have predisposing factors of either or both malignancy and chemotherapeutic agents, a logical association when one considers the volume of breast reconstruction and the inherent risk factors this population brings. Less commonly, PG can result from simple trauma to the body, termed traumatic PG. Typically, an innocuous injury lends itself to abnormal healing, a painful wound out of proportion to the injury, and the stereotypical stigmata associated with PG: red-violet wound edges, purulent discharge from the dermis and subcutaneous tissue, and a pathergic phenomenon, the worsening of the disease, and resulting wound with surgical debridement or mechanical disturbance. Approximately 30% of cases of PG exhibit pathergy.^5^ This pathergic phenomenon is often the difficult “trap” that causes problems with most surgeons. Even the most well-trained and educated surgeon is likely to have difficulty with an initial diagnosis of PG, likely exacerbating the pathergy with potentially exacerbating surgical debridement.

As compared with PG, wound infections are far more likely in a differential diagnosis list, given their frequency.^6^ The plastic and reconstructive surgeon is often asked for consultation for difficult and nonhealing wounds. A long list of causes for nonhealing wounds comes before arriving at a diagnosis of PG, as it truly is a diagnosis of exclusion. Not only is the clinical presentation almost identical to necrotizing infections but also there is no definitive pathologic diagnosis. There are suggestive dermatopathologic features, such as leukocytoclastic vasculitis, and later suppurative granulomatous dermatitis, but these are neither specific nor sensitive for this disease.^7^ This creates a diagnostic and ameliorative conundrum for surgeons.^8^ We present a case of a postoperative wound from a simple carpal tunnel release in a 33-year-old, right-hand-dominant, otherwise healthy woman. As the most commonly performed surgery of the hand and wrist annually, this is of keen interest to hand surgeons.^9^ We present the management, diagnostic workup, and outcome of this case. We then provide a recent literature review on the subject and a summary of PG in the surgical patient.

## METHODS

**Figure 1:**
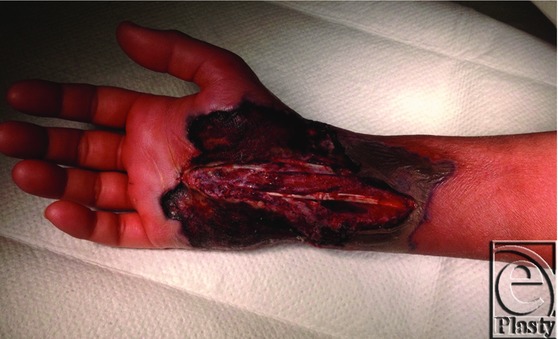
Initial wound prior to first operating room debridement. Significant necrotic tissue is noted at the edge of the wounds with concentric expansion from the centrally located original incision over the carpal tunnel.

**Figure 2:**
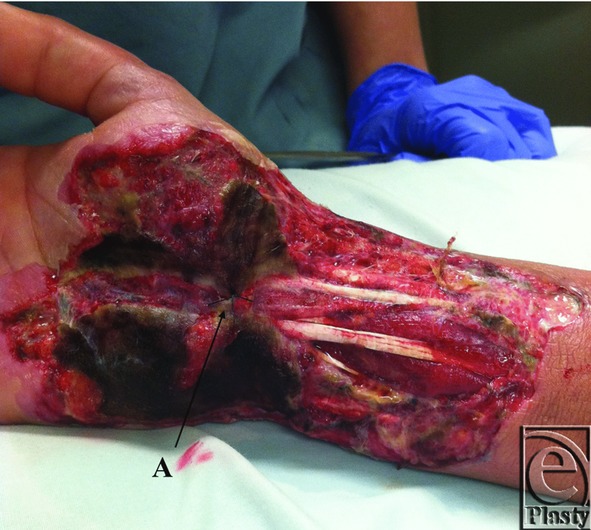
Initial presentation of the hand after 2 episodes of debridement at an outside facility. A: Single suture holding soft tissue over the median nerve to prevent desiccation. Note the significant necrotic debris without early appearance of a violaceous rim or frank purulence.

A 33-year-old woman presented to a rural hand surgeon for surgical consultation due to complaints of numbness and tingling in the median nerve distribution consistent with carpal tunnel syndrome. The patient was reportedly otherwise healthy aside from previous wound-healing complications following a cesarean section 4 years ago, for which she underwent a complicated abdominal wall reconstruction course after wound dehiscence. In addition, she noted a prolonged healing course of a traumatic leg wound as a child that healed without intervention. The patient was found to be a suitable candidate and was scheduled for open carpal tunnel release by the rural hand surgeon. The carpal tunnel surgery was uneventful, and the patient went home as an outpatient the same day. She represented to the hand surgeon on day 2 postoperatively with complaints of wound drainage and increased pain. The incision was opened proximally and distally and a Penrose drain was placed. The patient returned 3 days later after calling with complaints of worsening drainage, fever, and pain. The patient was seen in the emergency department, and it was determined that additional debridement should be undertaken (Fig 1). The patient underwent debridement that evening. The next day, additional necrotic tissue had developed, and it was determined that the patient should undergo additional debridement. This debridement resulted in exposed tendon and median nerve (Fig 2). The patient was transferred following this surgery for higher level of care and soft-tissue coverage.

A thorough history upon admission to our facility did demonstrate a history of necrotizing infections, including the aforementioned abdominal surgery, as well as an infection on her leg from a cut from a tree branch when she was an adolescent. This went on to heal without intervention over a prolonged period. The prevailing differential was limited mostly to necrotizing fasciitis or other microorganism infection, including fungal, protozoan, and viral. Given a negative history of immune disease, including a negative immunological workup, PG was lower on the differential list. Cultures were taken, and the patient underwent debridement operatively.

**Figure 3:**
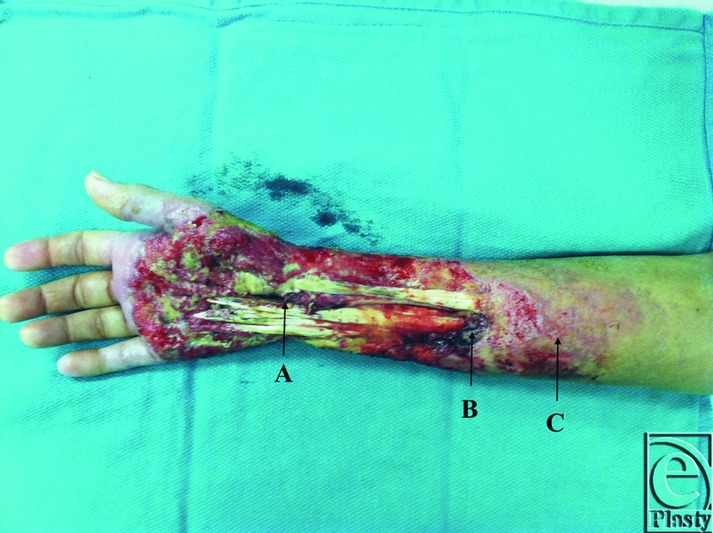
Wound after additional debridement of the enlarged wound. A: The median nerve is now exposed, as the flexor tendons are now devoid of paratenon and are no longer viable soft-tissue coverage. B: Necrosis noted at the musculotendinous junction. Subsequent debridement would result in dehiscence of the muscle and tendon at this level. There is also intradermal purulence visible. C: Appearance of violaceous periwound, which was previously not observed.

The patient developed additional necrosis of skin and vital structures over the next 5 days as bedside dressings were performed with Dakin's wet-to-dry dressings twice a day. The patient underwent additional debridement and temporization of the wound with allograft and wound vacuum-assisted closure (VAC) placement. Intraoperatively, the skin edges were noted to have a purple-red rim of tissue encircling the necrotic tissue, raising concern for vasculitis or PG (Fig 3, C). At this point, dermatology was consulted. It was at this time that a diagnosis of PG was established, as all cultures were negative and tissue pathology was nonspecific. The patient was started on prednisone 60 mg orally daily. The patient's condition began to improve after initiation of this treatment, but unfortunately with the first VAC change, allograft loss and additional necrosis of vital structures were noted. At this time, the patient noted no gross sensation in the median nerve distribution and minimal to no purposeful movement in the hand. The patient was transitioned to dapsone 5 days later in anticipation of long-term immunosuppression.

The patient returned to the operating room 1 day later for additional debridement, which revealed frank necrosis of an exposed median nerve at the mid-forearm level (Fig 3, A) and dehiscence of all flexor digitorum profundis and flexor carpi radialis tendons at the musculotendinous junction (Fig 3, B). Additional mechanical trauma was minimized at this time, removing only frankly necrotic material. Postoperatively, a second opinion from a senior partner was obtained regarding prognosis, ongoing management, and evaluation for mid-forearm amputation. It was agreed that because of a loss of motor function and sensation, along with the high risk associated with a free tissue transfer or separate donor site surgery, forearm amputation would provide the safest and best outcome for the patient.

## RESULTS

**Figure 4:**
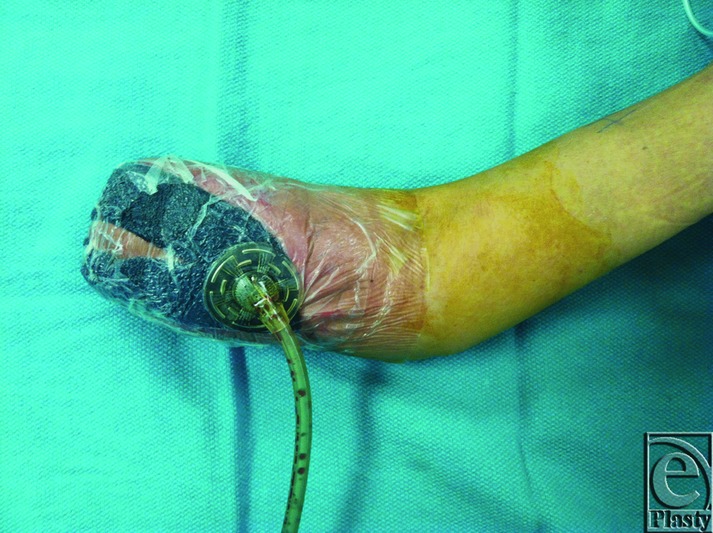
Affected arm after transradial mid-forearm amputation. Systemic immunosuppression was initiated prior to this procedure. VAC therapy was initiated and continued until the patient successfully healed this wound without further wounds or complication.

In light of additional surgical intervention, immunosuppression was optimized with the addition of cyclosporine 4 mg/kg as an adjunct to the dapsone and prednisone taper. The patient 3 days later underwent mid-forearm amputation, partial closure, and application of wound VAC (Fig 4). She was discharged from the hospital with a wound VAC approximately 1 week after her final surgery. The patient's post–hospital course has been relatively uneventful. She developed an additional lesion after discontinuation of cyclosporine, which was restarted shortly thereafter. Her wound healed completely approximately 1 month after initiation of immunosuppression and last surgical intervention. The patient's operative course is summarized in Table 1. The patient experienced 1 episode of remission after discontinuation of cyclosporine, which was reinitiated. This wound healed with dressing changes. The patient was weaned from immunosuppression after her wounds healed. She has not yet undergone an additional surgical procedure to evaluate for evidence of recurrence. The dermatology team recommended suppressive steroids prior to surgery to avoid recurrence or flare-up.

## DISCUSSION

The complications arising from delayed diagnosis and subsequent mismanagement of PG can be devastating, as noted in this case report. As surgeons, it is vital to know and understand this clinical entity in order to minimize the untoward effects it can have on patients. Plastic and reconstructive surgeons are uniquely positioned to encounter a large number of PG cases relative to other medical providers, as patients with difficult wounds, postsurgical or otherwise, often find themselves requiring our services.

The concept of PSPG is not a novel one, as it was first described in the early part of the 20th century by Brocq in 1916 and later redefined and renamed by Brunsting et al in 1930.^10^ A recent review looked closely at PSPG and found approximately 220 cases reported in the literature.^4^ While this is a far cry from a common occurrence, it is certainly more common than a single report in the literature. In addition, a disease such as this is likely to be grossly underreported in the literature, as it often goes misdiagnosed for long periods of time, results in numerous “unnecessary” surgical procedures, and may often eventually heal without any definitive intervention, leading to no true diagnosis. A study reported in the *Journal of Hand Surgery* in 2001 showed that the average case resulted in 2.2 unnecessary surgical procedures per patient for 7 patients presenting with PG lesions of the hand. Four of the 7 patients underwent amputation. The surgeon is not alone in this, though, as the average number of providers who had seen the patient prior to diagnosis was 5. The diagnosis was always made by or with the dermatology team.^11^

While the present case report demonstrates what is best classified as traumatic PG, it is important to note that a serologic workup for autoimmune disease is negative for this patient. In fact, approximately 50% of cases of PG are considered to be idiopathic. As a result, a clinical history of rheumatoid arthritis or inflammatory bowel disease may be absent, as it was in this case. Personal serologic testing may also be negative.^12^

Identification of risk factors is important in avoiding high-risk surgical patients likely to develop PG postoperatively. Unfortunately, this is often not feasible. As a result, awareness of the disease process as a possible outcome is perhaps the single most important factor in determining positive outcomes with the presentation of this disease. This is not to say that patient selection based on history and risk factors cannot play a role, but a surgeon cannot feasibly refuse to operate on a patient solely based upon the fact that he or she has risk factors for developing PSPG. Instead, a cautious approach should be undertaken.

In the patient scenario described in this case report, a number of factors contributed to the eventual amputation of the patient's arm. Incomplete patient history was perhaps one of the greatest contributing factors of delayed diagnosis, as knowledge of prior treatment with steroids and subsequent remission was not recognized until later in the patient's disease course.

In patients with a history of PG or PSPG, the clinician or surgeon should always elucidate the mode of previous treatment, its efficacy, and note whether the patient has had subsequent trauma or surgical procedures with this treatment in place. Commonly, patients with known PSPG can be placed on high-dose pulsed oral steroids prior to further procedures to safely avoid new manifestations of PG at the new surgical site (level IV evidence).^13^ Alternatively, Zakhireh et al^14^ described stabilization with cyclosporine prior to the treatment of pyoderma wounds with split-thickness skin grafts. While the former study looked at the ability to operate on separate sites in the future, the latter study utilized immunosuppression in combination with grafting to heal primary PG wounds.

The treatment of PSPG from a surgeon's perspective is quite simple in most cases. Once a definitive diagnosis is made, avoiding surgical debridement until the initial wounds are controlled with immunosuppressive therapy is paramount. The mainstays of medical therapy include prednisolone and cyclosporine dosed at 0.75 and 4 mg/kg per day, respectively, with a maximum dose at the 100-kg dose. A recent head-to-head comparison of these treatments demonstrated no difference in outcomes with monotherapy, including similar recurrence rates (30% and 28%, respectively) and adverse reactions (68% and 66%, respectively).^15^ It is important to maintain therapy for as long as 6 months following final surgical intervention, as the risk of recurrence with early termination of immunosuppression is high and was experienced in the present case.

There is increasing evidence in the literature for using biologic agents in the treatment of PG, including monocloncal antibodies, anti-TNFα agents, and intravenous immunoglobulin.^12^^,^^16^ TNFα and IL-1 have been strongly implicated in the destructive inflammatory process seen in PG. As a result, agents such as etanercept and ustekinumab are increasingly being used to control PG wounds.^16^

Another mainstay of therapy is VAC. A recent case series and review of the literature demonstrated successful closure of PSPG wounds following a mastopexy within 42 days without additional debridement.^17^ While the mechanism is not clear, VAC therapy appears to avoid the mechanical trauma responsible for the pathergic phenomenon noted with surgical debridement. There may even be a role for surgical debridement in the setting of concomitant hyperbaric oxygen^18^ and VAC therapies.^19^ In the case presented earlier, the patient experienced decreased wound progression and significantly better pain control after the initiation of VAC therapy. However, final closure was obtained with primary closure and incisional VAC therapy without the use of hyperbaric oxygen treatment. It is unclear whether there is a superior benefit to hyperbaric oxygen with VAC therapy compared with VAC therapy alone.

## CONCLUSION

Pyoderma gangrenosum is a rare but serious disease of particular importance to surgeons. Postsurgical pyoderma gangrenosum can have devastating consequences if early diagnosis is not confirmed and additional surgical debridement is undertaken. Early diagnosis is best accomplished with a keen awareness of this condition, a thorough history including investigation of previous nonhealing wounds and subsequent treatments, and early involvement of a medical and dermatology team, including a dermatopathologist. Early recognition and initiation of medical therapy are the key to avoiding progression of this aggressive wound-producing disease. Accepted therapies include steroids, immunosuppressive agents, and, more recently, monoclonal antibodies and biologic agents directed at the cytokines implicated in the disease's inflammatory process. It is crucial for the surgeon to avoid mechanical disruption and any other trauma to the dermal structures in this disease, as it will result in a pathergic phenomenon and worsening of the wound. Surgical debridement and treatment can often safely be undertaken once medical therapy is established. Subsequent surgical procedures can safely be performed with pulse-dose suppressive oral steroids. This is usually monitored and prescribed by the dermatology team, although a knowledgeable primary care physician or the current surgeon can manage this.

Failure to recognize PSPG early in the disease process can lead to numerous episodes of exacerbating debridement. While definitive diagnosis is difficult, owing especially to the fact that wound infection is much more commonly the cause of necrotic ulcers, awareness of this process may decrease the delay in diagnosis seen in many other cases. It is imperative that surgeons have knowledge of PG and PSPG and consider this when an early necrotizing wound appears in a postsurgical patient. Plastic surgeons, in particular, should be educated about this disease, given the association with breast and abdominal wounds. With excellent education, a high index of suspicion, and a multidisciplinary approach, patients who have the misfortune of developing PSPG may be able to avoid sequelae that include loss of function or form.

## Figures and Tables

**Table 1:** Operative dates relative to initial operation and establishment of postsurgical pyoderma gangrenosum diagnosis*

	Postoperative		
Event	day	Description of surgery/event	Indication
Initial carpal tunnel surgery	0	Limited incision open carpal tunnel release	Numbness and paresthesias
First postoperative visit	2	Suture removal and placement of the Penrose drain	Purulent drainage from the wound
Subsequent office visit	5	Bedside soft-tissue debridement	Increased wound drainage
OR debridement	6	Surgical debridement of the necrotic tissue	Frankly necrotic wound edges
Patient transferred^†^	6	N/A	N/A
First debridement following transfer	7	Debridement of soft tissue 100 cm^2^	Additional necrotic tissue
Second debridement^‡^	12	Irrigation and debridement of the right arm wound, placement of allograft 350 cm^2^, placement of wound VAC	Exposed vital structures and an increase in wound size
Final debridement	19	Excisional debridement of 100-cm^2^ right upper-extremity wound including the skin, muscle, tendon, and nerve	Significant desiccation of vital structures requiring debridement to prevent infection
Mid-forearm amputation	22	Mid-forearm amputation of the right arm through the radius and ulna	Exposed vital structures without viable functional recovery of the hand
VAC change	24	VAC change under sedation and steroid injection	Pain control with VAC change and intralesional steroid injection
VAC discontinued	33	VAC discontinued; wound management changed to wet-to-dry dressings alternating every week with Xeroform	Decreased wound size to 1.3 cm, seen in the office by the plastic surgery team
Wound healed	40	Dressings discontinued due to final wound healing; cyclosporine discontinued by the dermatology team	Wound no longer open; healed with primary closure and wound VAC
Additional wound formation	64	Additional wound noted by the patient; cyclosporine restarted by the dermatology team	Increased pain and pustule formation
Final healing	79	Date of final healing as determined by documentation of the healed wound	N/A

*OR indicates operating room; VAC, vacuum-assisted closure; and N/A, not applicable.

^†^The patient was transferred to higher level of care after 2 episodes of failed debridement with wound progression 6 days after initial carpal tunnel release.

^‡^Immediately following surgical debridement, dermatologic consultation was obtained, resulting in definitive diagnosis of pyoderma gangrenosum and initiation of appropriate steroid therapy.
